# Application of Ferriferous Oxide Modified by Chitosan in Gene Delivery

**DOI:** 10.1155/2012/920764

**Published:** 2012-12-27

**Authors:** Yu Kuang, Tun Yuan, Zhongwei Zhang, Mingyuan Li, Yuan Yang

**Affiliations:** ^1^West China School of Preclinical and Forensic Medicine, Sichuan University, Chengdu, Sichuan 610041, China; ^2^National Engineering Research Center for Biomaterials, Sichuan University, Chengdu, Sichuan 610064, China; ^3^ICU of West China Hospital, Sichuan University, Chengdu, Sichuan 610041, China

## Abstract

New approaches to improve the traditional gene carriers are still required. Here we explore Fe_3_O_4_ modified with degradable polymers that enhances gene delivery and target delivery using permanent magnetic field. Two magnetic Fe_3_O_4_ nanoparticles coated with chitosan (CTS) and polyethylene glycol (PEG) were synthesized by means of controlled chemical coprecipitation. Plasmid pEGFP was encapsulated as a reported gene. The ferriferous oxide complexes were approximately spherical; surface charge of CTS-Fe_3_O_4_ and PEG-Fe_3_O_4_ was about 20 mv and 0 mv, respectively. The controlled release of DNA from the CTS-Fe_3_O_4_ nanoparticles was observed. Concurrently, a desired Fe_3_O_4_ concentration of less than 2 mM was verified as safe by means of a cytotoxicity test in vitro. Presence of the permanent magnetic field significantly increased the transfection efficiency. Furthermore, the passive target property and safety of magnetic nanoparticles were also demonstrated in an in vivo test. The novel gene delivery system was proved to be an effective tool required for future target expression and gene therapy in vivo.

## 1. Introduction

Nonviral gene vectors have many advantages such as mass production, easier transportation, less immunogenicity, and being easily targeted to organs [1, 2]. Among the nonviral vectors, chitosan is known to possess efficient properties owing to their ability to condense nucleic acid into stable complexes, which protects DNA from degradation by nuclease [3]. The DNA/polymer complexes are taken up into the cells via endocytosis into the endosomes [4], following with burst release of complexes fraction in endosomes and the DNA translocates into the nucleus. Chitosan is copolymer of N-acetyl-glucosamine and glucosamine. It is soluble at acidic PH value, and the amino groups carry positive charge in acidic mediums; it can combine with negatively charged DNA. Moreover, chitosan also easily associates with iron oxide nanoparticles. It has been used generally in pharmaceutical applications [5]. Previous studies have revealed that chitosan, like other cationic polymers, displayed concentration-dependent toxicity toward cells in vitro, although it had many advantages as a gene vector [6].

Magnetic ferriferous oxide nanoparticles possess prominent advantages that might correct the defects of traditional drugs and gene carriers. They possess both magnetic and nanoeffects [7]. Whereby numerous DNA strands attached to the surface of these ferriferous oxides could reach the desired position with the help of static magnetic field. In order to improve the properties of nanoparticles such as biocompatibility, transfection efficiency, and controlled release, we embedded the biodegradable polymers on the surface of ferriferous oxide to form a core shell structure [8]. Therefore, the focus of our research was on how to improve the target property and remove the application barriers of nonviral gene vectors in vivo. The use of a static magnetic field has been shown to result in dramatic increase in transfection efficiency of gene delivery when compared with the conventional transfection system [9, 10]. Magnet-assisted transfection is a new, easy-to-handle, very highly efficient technology. It is a very gentle method with almost no toxicity and has been successfully used on many and also critical cell lines [11]. All types of nucleic acids from plasmid DNA or oligonucleotides to siRNA can be used with this approach [12]. In this research, the synthesized magnetic nanoparticles have an approximately size of 100 nm and are additionally coated with biodegradable polymers. We used both of the advantages of magnetic nanoparticles and biodegradable polymers, and the application of the novel polymer-Fe_3_O_4_ complexes as gene vectors in vitro was then described at length.

## 2. Materials and Methods

### 2.1. Preparation of Polymer-Fe_3_O_4_ Nanoparticles

The magnetic nanoparticles used as gene carriers are mostly iron oxides. These iron oxides can be generated by precipitation from acidic iron-salt solutions upon addition of appropriate bases [13]. Aqueous dispersions of Fe_3_O_4_ coated with polymers were prepared as latter. A CTS (MWs 45 kDa, 20% w/w, pH6.9) solution carrying a positive charge or PEG (MWs 6 kDa, 20% w/w) solution was prepared. 0.2 mL of this solution was added to 0.8 mL of iron oxide dispersion (10% w/w) for 8 h incubation. After filtration sterilization with a 0.45 *μ*m filter, the nanoparticles were used for the next transfection experiments. Nanoparticles and DNA form complexes by ionic interaction of the negatively charged nucleic acid and the positively charged surface of the CTS-Fe_3_O_4_ nanoparticle (N/P ratio 4 : 1). The polymer-Fe_3_O_4_ was analyzed by means of a transmission electron microscope (TEM, HITACHI H-700H), X-ray diffraction (XRD, Philips X'Pert PRO). The size and zeta potential of the polymer-Fe_3_O_4_ were both assessed using the Zetasizer Nano instrument.

### 2.2. Assay of DNA Encapsulation Efficiency

EGFP was used to monitor gene transfer and gene expression after transfection. The plasmid pEGFP-C1 was propagated in *Escherichia coli* and was purified using an Endotoxin-free Plasmid Maxiprep Kit (Qiagen). At the pH level of 7.4 the polymer-Fe_3_O_4_ complexes were mixed with DNA at different volume ratios in a 50 *μ*L reaction system. The final concentration (FC) of plasmid DNA and polymer Fe_3_O_4_ was 4 *μ*g/*μ*L and 1 mM (concentrations related to Fe) diluted with double-distilled water (ddH_2_O). After 1 h incubation at 37°C the concentration of DNA in the supernatant was measured by UV spectrophotometric absorption at 260 nm. The encapsulation efficiency (E.E.) of the process indicates the percentage of DNA encapsulated used for the preparation of polymer-Fe_3_O_4_ complexes.

### 2.3. Target Distribution of Polymer Fe_3_O_4_

To observe the target distribution of polymer-Fe_3_O_4_ nanoparticles in different organs of mice, 40 pathogen-free BALB/c female mice were purchased from the Sichuan Industrial Institute of Antibiotic for the in vivo studies. The polymer Fe_3_O_4_ was redispersed as described previously and injected through the caudal vein on the dosage of 1 mM iron oxide in 0.8 mL. A neodymium-iron-boron (NdFeB) permanent magnet (Br 1/4 1.5 T) was fixed to the surface of the extrahepatic skin for 6 hours. The mice were sacrificed at different times after the injection (2 h, 6 h, 12, and 24 h), and the liver, spleen, lungs, heart, and brain were taken out and made into tissue slices. The target distribution of polymer Fe_3_O_4_ was observed by Prussian blue and neutral red staining.

### 2.4. In Vitro Release

Release kinetics of plasmid DNA from magnetic nanoparticles were studied [14]. For this experiment, preweighed polymer-Fe_3_O_4_ complexes containing DNA were incubated in a test tube with phosphate-buffered saline (PBS, pH 7.4), for 30 min under moderate stirring at 37°C. DNA was reacted with polymer-Fe_3_O_4_ nanoparticles at three different volume ratios (1 : 3, 1 : 1, and 3 : 1). At predetermined time intervals (12, 24, 48, 72, and 96 h), 50 *μ*L of the released medium was collected by centrifugation (3,000 ×g, 1 min), and 50 *μ*L of fresh PBS was added back into the test tube. DNA release was monitored by UV spectroscopy at 260 nm, and DNA integrity was evaluated on a 1% agarose gel. The amount of released DNA was calculated from the free DNA concentration in the supernatants, and the curve of DNA release in vitro was described. At last, to confirm the functionality of released DNA, the discharged DNA was applied to the assay of transfection in vitro.

### 2.5. Test of DNaseI Treatment

The polymer-Fe_3_O_4_ complexes (1 mM) were mixed with plasmid DNA (4 *μ*g/*μ*L) according to the optimal E.E. Naked plasmid DNA and DNA/polymer-Fe_3_O_4_ complexes were incubated with or without DNaseI (0.5 U) in the 30 *μ*L reaction system for 1 hour at pH 7.4. The digestion was stopped by addition of 0.5 M EDTA. The product of enzymatic digestion was analyzed by 1% agarose gel electrophoresis, and DNA in the gel was visualized by ethidium bromide staining. Naked plasmid DNA after being digested by DNaseI and naked plasmid DNA without digestion were used as controls.

### 2.6. Cell Culture and Cell Viability Assay

Human Embryonic Kidney 293 cells (HEK-293), human liver carcinoma cells (HepG2), and mouse myeloma cell line (SP2/0) were maintained in DMEM or RPMI-1640 medium (Gibco-BRL), supplemented with 10% fetal calf serum (FCS, Gibco-BRL) and 1% penicillin/streptomycin. For the transfection and cytotoxicity test, the cells were grown under standard conditions for 24 hours until 70% to 80% confluency in 96-well flat-bottomed microassay plates before the addition of either the plasmid DNA/polymer-Fe_3_O_4_ complex or only the polymer Fe_3_O_4_.

Assessment of cell viability was performed by the MTT assay. Firstly, the precipitate polymer-Fe_3_O_4_ complexes were resuspended under conditions of ultrasonic agitation for 10 min. Subsequently, the complexes were added into the cell-culture fluid at a different concentration (0.2 ~ 1.0 mM, 2 ~ 20 mM), diluted with a serum-free medium. At the end of each predetermined time (6 h, 12 h, 24 h, and 48 h), the polymer-Fe_3_O_4_ complexes were replaced with 200 *μ*L of fresh DMEM medium. Then, 20 *μ*L of MTT (5 *μ*g/*μ*L) in DMEM was added to each well and incubated for an additional 4 hours. All mediums were then removed, and 150 *μ*L of DMSO was added to dissolve the crystals formed by the live cells. Absorbance was measured at 570 nm using a Bio-Tek EL-311microplate reader. The cell viability was calculated, and the viability of nontreated control cells was arbitrarily defined as 100%.

### 2.7. Magnet-Assisted Transfection In Vitro

24-well plates were seeded with 2 × 10^5^ cells (HepG2 and SP2/0 cells) and grown for 24 hours to obtain 70–80% confluence. Prior to transfection, the medium was removed, and the cells were rinsed once with PBS (pH 7.4), then supplied with serum-free medium. The plasmid DNA was mixed with CTS-Fe_3_O_4_ and PEG-Fe_3_O_4_ as described previously and incubated for 30 minutes at 37°C. DNA/polymer-Fe_3_O_4_ complexes were suspended in a serum-free medium to get the final concentrations of 2 *μ*g/*μ*L and 1.5 mM, respectively. To verify the short exposure to a static magnetic field would improve transfection efficiency; the cells were placed on a (NdFeB) magnet for 30 min at a distance of 3 mm from the magnet surface, which leads to a magnetic flux density of 340 mT and a magnetic field gradient perpendicular to the well plate of 14 T/m. After a further incubation of 4 h, the medium was removed and a new medium containing 10% FCS was added. The cells were incubated with plasmid DNA alone and DNA/polymer-Fe_3_O_4_ complexes under standard conditions and grown in culture medium for 24 hours to allow for EGFP expression. Concurrently, transfection was performed using nonmagnetic transfection reagents. Chitosan (MWs 45 kDa), lipofectamine (BestBio), and PBS were added to an equal volume of DNA as controls. Transfected cells expressing green fluorescent protein were detected using a Leica fluorescence microscope.

## 3. Results and Discussion

### 3.1. Characteristics of Polymer-Fe_3_O_4_ Nanoparticles

**Figure 1:**
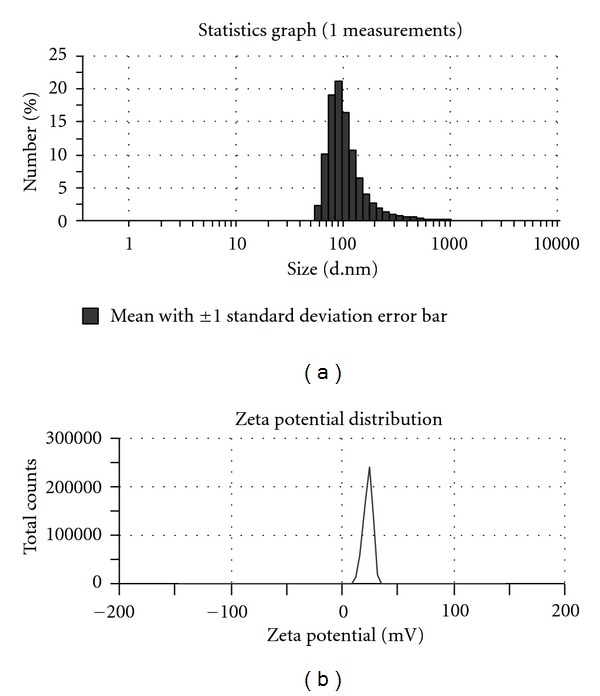
The size and zeta potential of the CTS-Fe_3_O_4_. (a) Size of distribution of the CTS-Fe_3_O_4_; (b) zeta potential of the CTS-Fe_3_O_4_.

TEM images showed that most of the iron oxide complexes were approximately spherical (unpublished data). The XRD measurements also indicated that the samples had a cubic crystal system and magnetite Fe_3_O_4_ was the dominant body of the polymer-Fe_3_O_4_ complexes. The size and zeta potential showed the two samples to have a uniform size of 100 nm (Figure 1(a)) and almost the same distribution. The sizes of 10–100 nm in diameter are desirable since they are too small not to be eliminated by the reticuloendothelial system (RES) but too large to be filtered out by the kidneys [15]. CTS-Fe_3_O_4_ had a positive charge of about 20 mv (Figure 1(b)), and the zeta potential of PEG-Fe_3_O_4_ was 0 mv. It has been reported that surface charge plays an important role in determining the efficiency and mechanism of cellular uptake [16]. It is also an important factor to improve stability of polymer-Fe_3_O_4_ complexes and to prevent from further aggregation in aqueous solution via electrostatic repulsion [17]. Zata potential value showed the main binding ability between the polymer Fe_3_O_4_ and DNA. The polymer-Fe_3_O_4_ complexes were mixed with plasmid DNA according to different volume ratios (1 : 3, 1 : 2, 1 : 1, 2 : 1, and 3 : 1) in a 50 *μ*L reaction system. It was obvious that the E.E. increased along with the proportion of the magnetic materials mainly because of the electrostatic interactions, surface energy of nanoparticles, and branched structures of polymers. The optimal E.E emerged when the iron oxide complexes were mixed with DNA at 3 : 1 volume ratio, and the final concentration of DNA and iron oxide was 2 *μ*g/*μ*L and 1.5 mM respectively. The concentration corresponded with the transfection and cell viability assay latter. In addition, the E.E. of PEG-Fe_3_O_4_ was inferior to CTS-Fe_3_O_4_ notably for the lack of electrostatic attraction.

### 3.2. Target Distribution In Vivo

**Figure 2:**
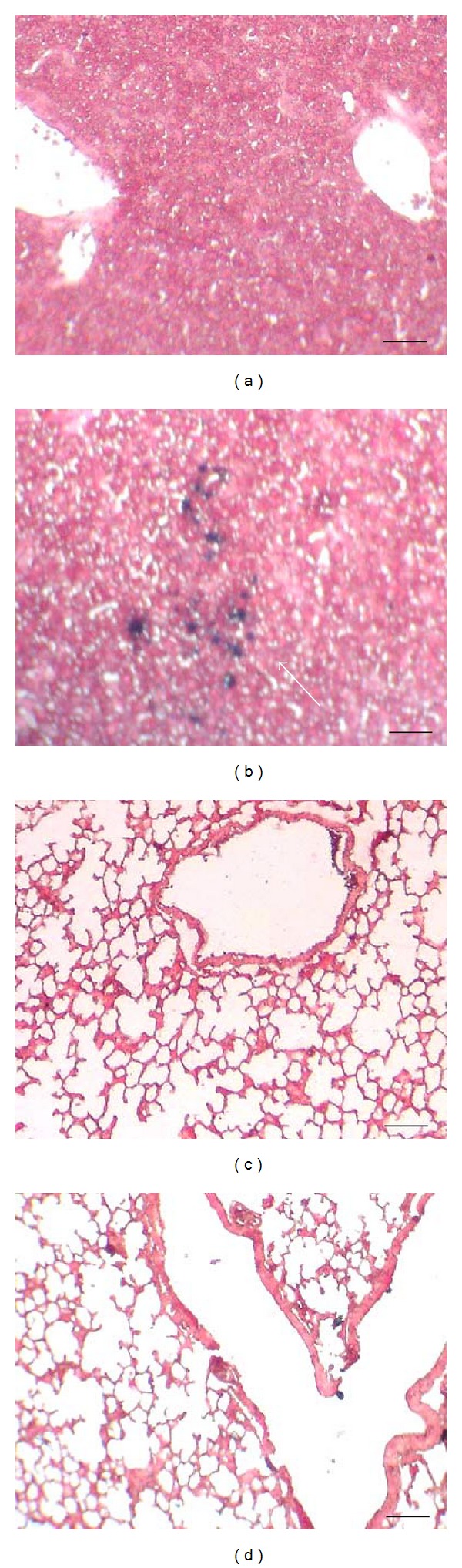
Target distribution of magnetic CTS-Fe_3_O_4_ in liver and lung tissue. Figures were shown by Prussian blue and neutral red staining (×250), with outer static magnetic field for 2 hours. (a) Normal liver tissue; (b) liver tissue injected CTS-Fe_3_O_4_ nanoparticles (1 mM); (c) normal lung tissue; (d) lung tissue injected CTS-Fe_3_O_4_ nanoparticles (1 mM). Scale bars correspond to 10 *μ*m.

The different organs from the mice injected with polymer-Fe_3_O_4_ were taken out and made into tissue slices. Target distribution of polymer Fe_3_O_4_ in vivo was demonstrated with the help of outer static magnetic field.  Figure 2(b) shows a large number of iron particles scattered in the hepatic tissue; many of them were distributed along the hepatic sinusoid 2 h after injection. The iron particles decreased gradually over time and disappeared 24 h after injection (data not shown). The shape of the liver cells was seen under a high-power microscope to be integrated. There was no iron staining in the other organs, such as the lungs (Figure 2(d)), the spleen, and the heart. And there was no obvious side effect observed in the injected mice.

### 3.3. Test of Polymer-Fe_3_O_4_-Loaded DNA In Vitro

Protection of DNA from DNaseI degradation was detected by 1% agarose gel electrophoresis. Naked pEGFP-C1 without digestion and naked pEGFP-C1 following digestion by DNaseI were used as controls. We could evidence partial protection of DNA coated by polymer Fe_3_O_4_ from nuclease-mediated DNA degradation (unpublished data). It was assumed that DNA degradation occurs in several layers; external layers will be degraded easily but not internal layers. Furthermore, CTS-Fe_3_O_4_ nanoparticles offered higher protection for DNA than PEG-Fe_3_O_4_, as the DNA chains could be attached more strongly to the former. In addition, DNaseI digestion resulted in a shift in the most distribution of the DNA isoforms: supercoiled plasmid in nontreated samples was replaced by the open loop form in treated samples.

**Figure 3:**
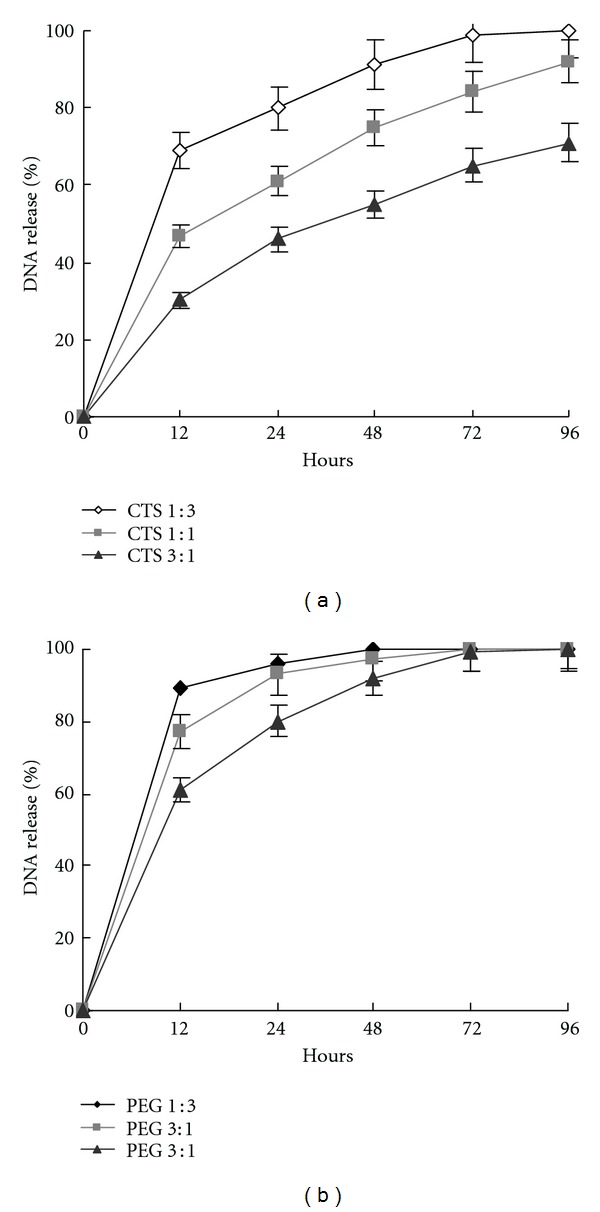
Kinetics of DNA release from the magnetic nanoparticles in vitro. (a) Percentage of DNA release coated by CTS-Fe_3_O_4_ and (b) percentage of DNA release coated by PEG-Fe_3_O_4_ at PH 7.4. The data shown are the mean ± standard deviation for three independent experiments.

The in vitro release rates of DNA from polymer-Fe_3_O_4_ complexes were studied at different volume ratios. A significant proportion (30%) of the adsorbed DNA was released very rapidly from the CTS-Fe_3_O_4_ nanoparticles in the initial 12 hours. After 48 h, the amount of released DNA reached 55% at the optimal E.E. And the remainder of the adsorbed DNA was released slowly, reaching 70% at 96 h (Figure 3(a)). Compared to DNA release from CTS-Fe_3_O_4_, a burst release phase of more than 61% from PEG-Fe_3_O_4_ was observed. The release curve showed that the DNA was released more rapidly; more than 80% of DNA was discharged from PEG-Fe_3_O_4_ after 24 h at the optimal E.E., and the entire release was mostly completed at 72 h (Figure 3(b)). The DNA integrity test at predetermined time points was assessed by agarose gel electrophoresis (data not shown). No differences were observed between EGFP expression from the released DNA and the controlled plasmid pEGFP-C1, indicating that adsorption and release from the polymer-Fe_3_O_4_ do not alter the functionality of plasmid DNA. Overall, the controlled release effect of CTS-Fe_3_O_4_ complexes was relatively obvious compared with PEG-Fe_3_O_4_. The speed of DNA release was inversely proportional to the volume ratios of nanoparticles. 

The N/P ratio (the ratio of negatively charged DNA to positively charged chitosan) is a key factor to determine the optimal complexation conditions. The difference PH and counterions in the medium might directly affect the binding between CTS and DNA [18]. It could be inferred that the burst release was induced by the DNA degradation in the external layers. The results showed that the controlled-release effect of CTS-Fe_3_O_4_ was more obvious, and the unsteady binding power made the efficient binding with DNA and PEG-Fe_3_O_4_ impossible. In addition, the small proportion of chitosan in the polymer-Fe_3_O_4_ complexes actually hindered the effect of controlled release. Increasing the proportion of chitosan would slow down the DNA release but augment the particle size and positive charge of the complexes. It has been reported that positively charged nanoparticles exhibited dose-dependent hemolytic activities and cytotoxicities [19]. In addition, most of the larger nanoparticles (>150 nm) are trapped by the liver and lung where many macrophages are located [20]. For the drug and gene target delivery application, the nonspecific uptake of nanoparticles by macrophages in the RES should be minimized. The contradictory issue of controlled-release and particle size needs to be resolved urgently by carrying out a further study.

### 3.4. Cell Viability and Magnet-Assisted Transfection

Low cytotoxicity is one of the major requirements for nonviral vectors for gene delivery. Chitosan was chosen as a functionalizing polysaccharide because of its biocompatibility. It has been reported that chitosan derivatives are less toxic than other cationic polymers such as PEI in vitro and in vivo [21]. Evaluation of cell viability was conducted on HEK-293 and HepG2 cells using a 0.2–20 mM concentration gradient of polymer-Fe_3_O_4_ complexes for different incubation periods. More than 90% cell viability of both polymer-Fe_3_O_4_ complexes was obtained after 24 h of incubation with a concentration of 2 mM or less, and apparent cytotoxicity emerged when the concentration of polymer Fe_3_O_4_ was more than 10 mM (data not shown). This result showed that both CTS-Fe_3_O_4_ and PEG-Fe_3_O_4_ had low cytotoxicity. There was no significant difference in cytotoxicity between the two kinds of magnetic materials. The security application could therefore be deduced according to the previously mentioned data and the optimal E.E.

**Figure 4:**
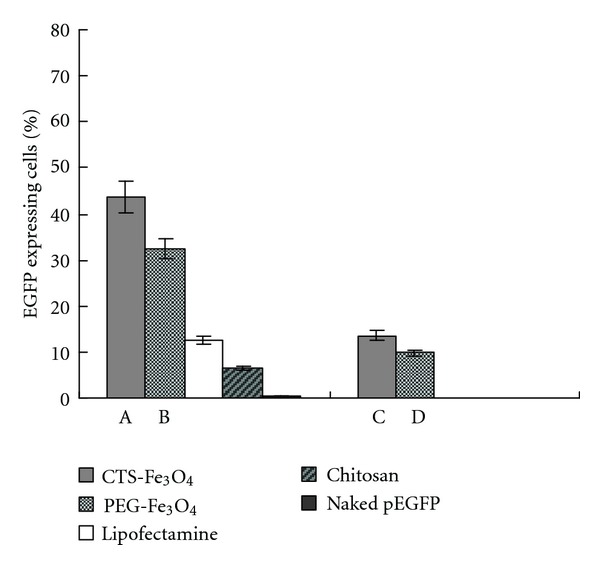
Magnet-assisted transfection of pEGFP plasmid. The SP2/0 cells were transfected with either polymer Fe_3_O_4_ or traditional transfection methods in the presence or absence of static magnetic field for 30 min. A and B: magnet-assisted transfection; other groups: traditional transfection. Data are shown as means and SD values from at least three independent experiments (*P* < 0.01 between A and C; B and D).

HepG2 and SP2/0 cells were transfected as described previously with either DNA/CTS-Fe_3_O_4_ or DNA/PEG-Fe_3_O_4_, with DNA/chitosan, DNA/lipofectamine, and naked plasmid as controls. Exposure to a permanent magnetic field (magnet) for 30 min was followed by 4 h incubation. Concurrently, the control groups were routinely transfected using conventional methods. The highest transfection rates were achieved in HepG2 cells corresponding to 67.2% and 45.8% after transfected with CTS-Fe_3_O_4_ and PEG-Fe_3_O_4_ complexes. Significantly lower transfection rates of 14.3%, 8.7%, and 0.4% resulted from transfection with lipofectamine, chitosan, and naked plasmid, respectively. In addition, the transfection rates were significantly increased by 4.1- and 3.2-fold in HepG2 and SP2/0 cells, when compared to cells not exposed to the magnetic field. Similar transfection results were also obtained with SP2/0 cells, and lower rates of 43.7% and 32.5% treated with CTS-Fe_3_O_4_ and PEG-Fe_3_O_4_ complexes were achieved. Compared with conventional transfected methods, the results were still statistically significant (Figure 4). Thus, the transfections rates enhanced by the assistance of magnetic field were verified again in HepG2 and SP2/0 cells. It seems that the use of a static magnetic field can improve the translocation of the particles across the cell membrane. It has been reported that the higher transfection rates with magnetic nanoparticles were mainly attributed to their size surface charge, since the larger nanoparticles faster sedimentated on the surfaces of the cells, and this resulted in higher endocytic uptake, and positively charged nanoparticles were more easily taken up by cells [22]. The chosen cells used in our study were malignant cells from human and mice, and these cells differed in characteristics used as models for different human diseases. Thus, they were good representative samples for enhancement of delivery and effective targeting of gene expression. Furthermore, the EGFP expression was strong in transfected cells indicating that the function of DNA was kept and no fragmentation occurred.

Magnetic materials modified by biodegradable polymers as gene carriers possess many merits. For examples, simple manufacturing operation, arriving at the target point with the help of an outer magnetic field; a powerful surface energy effect and a small size effect are their outstanding characters. Moreover, it is easy to modify all kinds of multifunctional groups or targeting molecules to form the structure of the core shell, such as CTS, PEI, specific ligands, and monoclonal antibodies, since the complexes have multiple binding sites on their surface, and DNA attaches itself to them in sizeable amounts either through an electrostatic effect or by chemical bond coupling. In order to improve the E.E. of the polymer-Fe_3_O_4_ complexes and realize the controlled release of the DNA, we modified the Fe_3_O_4_ with multifunctional groups CTS and PEG. In addition, the process of linking polymeric groups did not utilize organic solvent extraction, and the iron content used does not surpass the acceptable daily intake. Furthermore, some of the novel nanoparticles could improve the antigen presentation effect, show a better adjuvant effect, and make a long-term, single-immunization vaccine possible [23]. There are likely to be further applicative studies of polymer-Fe_3_O_4_ complexes as gene delivery systems. Preliminary data from our studies suggest that Fe_3_O_4_ nanoparticles when decorating with positive-charged polymer CTS exhibit preferential gene delivery.

## 4. Conclusion

CTS-Fe_3_O_4_ and PEG-Fe_3_O_4_ were successfully prepared. DNA encapsulation efficiency increased with the proportion of polymer-Fe_3_O_4_ nanoparticles, and the optimal E.E. (3 : 1) was chosen. Simultaneously, the attachment of DNA to polymer-Fe_3_O_4_ complexes did provide protection against cleavage by nuclease. The target distribution of polymer-Fe_3_O_4_ complexes with an outer magnetic field was demonstrated in vivo. The controlled-release effect of CTS-Fe_3_O_4_ complexes was more apparent than PEG-Fe_3_O_4_, and the DNA binding and release from the polymer-Fe_3_O_4_ do not alter its functionality. Both CTS-Fe_3_O_4_ and PEG-Fe_3_O_4_ had low cytotoxicity to HEK-293 and HepG2 cells. The concentration of 2 mM or less in an in vitro application was shown to be absolutely safe. In addition, the magnetic forces lead to an accelerated sedimentation of polymer-Fe_3_O_4_ complexes on the cell surface and do directly enhance the transfection efficiency in HepG2 and SP2/0 cells compared with conventional transfection methods. The novel gene delivery system proved to be an effective tool for future, and it is promising in targeting expression and delivery of therapeutic genes in in vivo studies. Our study explored the application of polymer-Fe_3_O_4_ nanoparticles as gene carriers. We will continue to do research in this field to provide a more detailed evaluation about the transfer of DNA.
